# Angiovolume and Peak Enhancement on Preoperative CAD-Derived MRI as Prognostic Factors in Primary Operable Triple-Negative Breast Cancer

**DOI:** 10.3390/tomography11120137

**Published:** 2025-12-05

**Authors:** Bo La Yun, Sun Mi Kim, Sung Ui Shin, Su Min Cho, Yoon Yeong Choi, Mijung Jang

**Affiliations:** Department of Radiology, Seoul National University Bundang Hospital, Seoul National University College of Medicine, 82, Gumi-ro 173 Beon-gil, Bundang-gu, Seongnam-si 13620, Gyeonggi-do, Republic of Korea; yunbola@gmail.com (B.L.Y.); kimsmlms@daum.net (S.M.K.); shinsungui@gmail.com (S.U.S.); sumin943@gmail.com (S.M.C.); y2choi822@gmail.com (Y.Y.C.)

**Keywords:** triple negative breast neoplasms, breast neoplasms, magnetic resonance imaging, diagnosis, computer-assisted, disease-free survival

## Abstract

**Background/Objectives:** To identify preoperative MRI features using computer-assisted diagnosis (CAD) that are associated with invasive disease-free survival (IDFS) and distant metastasis-free survival (DDFS) in patients with primarily operable triple-negative breast cancer (TNBC). **Methods:** This retrospective study was approved by the institutional review board with informed consent was waived. Between January 2012 and December 2014, 74 consecutive women with primary TNBC (mean age, 51 years; range, 29–77 years) who underwent preoperative MRI were included and followed until August 2021. Dynamic contrast-enhanced and T2-weighted images were obtained using 3T scanners. Peritumoral edema and central necrosis were evaluated retrospectively. CAD was used to extract 3D diameters, angiovolume, and kinetic parameters, and kinetic heterogeneity was calculated. Cox proportional hazards models were used to assess associations between MRI features and IDFS and DDFS, adjusting for clinicopathologic factors. **Results:** During a median follow-up of 80.9 months, 12 patients developed invasive disease, and 8 developed distant metastasis. In multivariable analysis, peak enhancement (hazard ratio [HR], 1.40; 95% confidence interval [CI], 1.06–1.84; *p* = 0.019) and angiovolume (HR, 2.86; 95% CI, 1.26–6.47; *p* = 0.012) were independently associated with IDFS, whereas angiovolume (HR, 2.47; 95% CI: 1.28–4.78; *p* = 0.007) was independently associated with DDFS. **Conclusions:** Preoperative CAD-derived MRI features, particularly peak enhancement and angiovolume, were associated with IDFS in TNBC patients whereas angiovolume alone was associated with DDFS.

## Simple Summary

This study explored preoperative MRI biomarkers that predict recurrence in triple-negative breast cancer (TNBC). Using computer-assisted diagnosis, we quantified peak enhancement and angiovolume as indicators of tumor vascular characteristics. Larger angiovolume and higher peak enhancement were associated with worse survival outcomes. These MRI-derived features may help identify high-risk TNBC patients and guide personalized treatment strategies.

## 1. Introduction

The triple-negative breast cancer (TNBC) is an aggressive subtype of breast cancer characterized by the absence of estrogen receptors (ER), progesterone receptors (PR) and human epidermal growth factor receptor type 2 (HER2) overexpression [1]. Among breast cancer subtypes, TNBC has the worst prognosis and is associated with more aggressive histological features [2,3]. Adjuvant or neoadjuvant chemotherapy is recommended for most patients with early-stage TNBC [4]. Although approximately 80% of TNBCs are basal-like breast cancers, TNBC is heterogeneous, sharing only the absence of ER, PR and HER2 expression.

TNBC patients have a higher recurrence rate within the first 1–3 years after diagnosis compared to non-TNBC patients. However, beyond this period, their recurrence rate becomes comparable to that of non-TNBC patients [3,5]. Genomic and transcriptome studies have further classified TNBC into seven molecular subtypes. Among these, basal-like subtypes and mesenchymal subtypes are generally associated with worse prognosis, while immunomodulatory subtypes tend to show better outcomes [6,7,8]. The presence of tumor-infiltrating lymphocytes is a maker of better prognosis [9,10]. Similarly, patients who achieve a pathologic complete response after neoadjuvant chemotherapy have excellent survival rates [5]. In contrast, biomarkers such as CK 5/6 or EGFR expression in TNBC have shown variable prognostic associations, reflecting the complexity of TNBC [11,12,13]. Despite these efforts, no internationally accepted prognostic marker has been established to guide treatment intensity or follow-up strategies in TNBC.

Dynamic contrast-enhanced (DCE)-MRI is widely used in preoperative breast cancer assessment and provides both morphologic and kinetic information about breast cancer. Through computer-assisted diagnosis (CAD) systems, pixel-based kinetic and volumetric features can be reproducibly extracted. Several studies have demonstrated correlations between CAD-derived MRI features and tumor subtypes, histologic grade, and Ki-67 [14,15,16]. Moreover, some preoperative MRI features such as higher peak enhancement, washout kinetics, increased ipsilateral vascularity, and higher positive skewness in texture analysis have been linked to worse disease-free survival in breast cancer. Higher kinetic heterogeneity and peak enhancement were associated with distant metastasis-free survival (DDFS) [15,17,18,19,20]. However, these findings largely encompass all breast cancer subtypes, and evidence specific to TNBC imaging is limited [21,22].

The purpose of this study was to identify preoperative MRI features derived from CAD that are associated with invasive disease-free survival (IDFS) and DDFS in patients with primary operable TNBC.

## 2. Materials and Methods

This retrospective study was approved by the Institutional Review Board of our hospital (IRB No. B-1511-322-104), and the requirement for informed consent was waived.

### 2.1. Study Population

Between January 2012 and December 2014, medical records at our institution identified 1420 consecutive women newly diagnosed with invasive breast cancer by core needle biopsy who underwent preoperative breast MRI including DCE imaging, followed by surgery. Among these, 143 patients were confirmed to have TNBC based on immunohistochemistry (IHC). ER, PR, and HER2 status were obtained from the surgical pathology reports. A sample was considered negative for ER or PR if less than 1% or 0% of tumor cell nuclei were immuoreactive [23]. HER2 expression was scored as 0, 1+, 2+, or 3+ based on IHC. Tumors with scores of 0 or 1+ were considered HER2-negative, while those with a score of 2+ underwent fluorescence in situ hybridization for confirmation.

**Figure 1:**
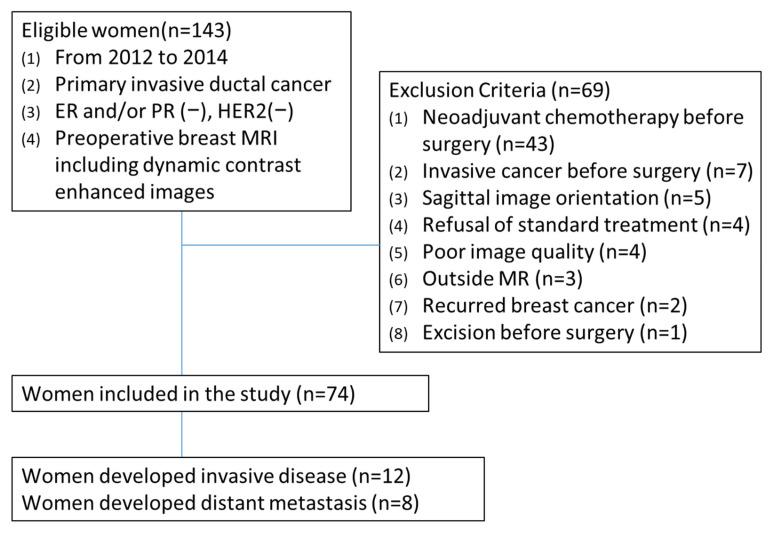
Patient selection flowchart.

Sixty-nine women were excluded for the following reasons: (a) recipient of neoadjuvant chemotherapy (*n* = 43), (b) history of another invasive cancer (*n* = 7), (c) sagittal dynamic MRI acquisition (*n* = 5), (d) refusal of standard treatment (*n* = 4), (e) poor image quality (*n* = 4), (f) MRI performed at outside facilities (*n* = 3), (g) recurrent breast cancer (*n* = 2), and (h) prior excision before surgery (*n* = 1). For patients with multiple tumors, only the largest lesion was analyzed. Finally, 74 women with 74 triple-negative invasive breast cancers were included (Figure 1). All MRI examinations were performed after the initial breast cancer diagnosis.

### 2.2. MRI Acquisition

All examinations were performed using a 3.0-T MR system (Achieva or Ingenia 3.0T, Philips Medical Systems, Best, The Netherlands) with a dedicated breast coil. Patients were imaged in a prone position. The protocol included turbo spin-echo T2-weighted imaging (T2WI) and 3D gradient-echo T1-weighted imaging (T1WI) with fat suppression using spectral attenuated inversion recovery (SPAIR).

Axial fat-saturated T2WI was obtained (repetition time [TR]/echo time [TE], 4788–8989/70–120 ms). DCE-MRI was performed after intravenous injection of gadobutrol (Gadovist; Bayer Schering Pharma, Berlin, Germany) at a dose of 0.1 mmol/kg body weight (injection rate, 1.5 mL/sec; 20 mL saline flush). For DCE-MRI, the acquisition parameters were as follows: on the Achieva system, TR = 4.0 ms, TE = 2.0 ms, slice thickness = 2 mm, flip angle = 10–12°, field of view (FOV) = 340 × 340 mm, matrix = 376 × 378, and echo train length (ETL) = 58; on the Ingenia system, TR = 6.0 ms, TE = 3.0 ms, slice thickness = 2 mm, flip angle = 12°, FOV = 340 × 340 mm, matrix = 576 × 576, and ETL = 30.

The interval between MRI and surgery ranged from 1 to 31 days (median, 8 days). Axial DCE-MRI consisted of one pre-contrast and five post-contrast series, with a temporal resolution of 60–90 s depending on breast size. The second post-contrast series was defined as the peak phase, and the fifth as the delayed phase.

### 2.3. MRI Analysis

On T2WI, peritumoral edema and intratumoral high signal intensity (SI) were assessed in consensus by two breast radiologists (with 10 and 13 years of experience in breast imaging), blinded to the clinical information [24]. CAD features were retrospectively extracted by one radiologist using a commercial system (CADstream, version 4.1.3; Confirma, Kirkland, WA, USA).

Pre-contrast and five post-contrast T1WI were analyzed. Relative to the pre-contrast baseline images, a pixel value increase of 50–100% at the peak post-contrast series was classified as medium enhancement, while an increase of more than 100% was classified as rapid enhancement. A color map was generated according to the delayed enhancement type following peak enhancement. Enhancement was categorized into three patterns: persistent, defined as a pixel SI increase of ≥10% in the delayed series compared with the peak series; plateau, defined as a pixel SI change of <10% (increase or decrease) between the delayed and peak series; and washout, defined as a pixel SI decrease of ≥10% in the delayed series relative to the peak series.

Following the reviewer’s click on the lesion within the color map, the software automatically segmented the entire lesion volume. Subsequently, the software calculated the volumetric percentages of medium persistent, medium plateau, medium washout, rapid persistent, rapid plateau, and rapid washout enhancement. A size adjustment feature was available when the segmentation was deemed inadequate by the reviewer. This function permitted the modification of the segmented area’s size by including or excluding surrounding regions in the color map, explicitly without adjusting the enhancement threshold. The software also provided orthogonal diameters, peak enhancement (maximum percentage increase), angiovolume (volume of the enhancement color map), and tumor volume (calculated using the ellipsoid formula). The maximum 3D diameter was defined as the longest among the orthogonal diameters. Kinetic heterogeneity was calculated as previously described [18].

### 2.4. Clinicopathologic Data and Follow-Up

Clinical information was collected from chart review, including age at surgery, interval between MRI and surgery, type of breast surgery, type of axillary dissection, use of chemotherapy and radiotherapy, as well as the time and site of first locoregional progression, subsequent metastatic progression, and last follow-up. Pathologic data included tumor size; histologic grade (1–2 vs. 3); presence of lymphovascular invasion; lymph node metastasis; Ki-67 index (≤20% vs. >20%); and expression of p53, CK5/6, and EGFR. Tumors were classified as basal-like if positive for CK5/6 or EGFR.

Patients received adjuvant chemotherapy and radiotherapy after surgery according to National Comprehensive Cancer Network guidelines [25]. Surveillance for locoregional or contralateral breast recurrence and distant metastasis was conducted in accordance with our institutional practice, with all patients undergoing annual breast ultrasound and mammography, chest radiography, bone scintigraphy, chest CT, and abdominal ultrasound. Data on survival and recurrence status were extracted from the patients’ medical and imaging records.

### 2.5. Statistical Analysis

The primary outcomes were IDFS and DDFS. The definition of IDFS included invasive locoregional recurrence (limited to the ipsilateral breast or chest wall and/or axillary, supraclavicular, or internal mammary lymph nodes) and distant recurrence (metastasis to other parts of the body), as well as contralateral breast cancer, second non-breast primary cancer, and death from any cause. DDFS was defined as distant recurrence, second non-breast primary cancer, and death from any cause, excluding invasive locoregional recurrence and contralateral breast cancer [26]. The last date of follow-up was 20 August 2021. Patients without events or lost follow-up were censored. To assess potential inter-scanner variability, quantitative CAD parameters were compared between the two MRI systems (Achieva vs. Ingenia) using the Student’s *t*-test for continuous variables and Fisher’s exact test for categorical variables. Cox proportional hazards models were used to assess the associations between MRI features and IDFS or DDFS, adjusting for clinicopathologic features. Hazard ratios (HRs) and 95% confidence intervals (CIs) were calculated. HRs for continuous variables were scaled to a 100% increase in peak enhancement, a 5 mL increase in angiovolume, and a 5 cm^3^ increase in tumor volume. Variables with *p* < 0.05 in univariate analysis were entered into multivariate models. Kaplan–Meier survival curves were generated for significant variables, with cut-off values for continuous variables determined using maximally selected log-rank statistics, and differences between groups were assessed using the log-rank test. Statistical analyses were performed using STATA/SE 14.0 (StataCorp, College Station, TX, USA) and R version 4.5.2 (R Foundation for Statistical Computing, Vienna, Austria) for Firth’s penalized Cox regression. A two-sided *p* < 0.05 was considered statistically significant.

## 3. Results

### 3.1. Patient Characteristics and Outcomes

Clinicopathologic characteristics of the 74 patients are summarized in Table A1. The mean age at surgery was 51 years (range, 29–77 years). Sixty-two patients (83.8%) underwent breast-conserving surgery (BCS), and all received adjuvant radiotherapy. All TNBCs were invasive ductal carcinomas. The mean pathologic tumor size was 1.8 cm (range, 0.4–5.3 cm), and axillary lymph node metastasis was present in 12 patients (16.2%). High histologic grade (grade 3) was observed in 59 patients (79.7%).

The median follow-up was 80.9 months (range, 3.4–113.3 months) for IDFS. During follow-up, 12 patients developed invasive cancers: two had ipsilateral breast recurrence, two developed contralateral breast cancer, and eight (10.8%) developed distant metastasis at a median follow-up of 81.9 months (range, 3.4–113.3 months). The lung and distant lymph nodes were the most common metastatic sites, followed by bone. Among the eight patients with distant metastasis, five died, with a median follow-up of 82.8 months (range, 3.4–113.3 months). There were no significant differences in quantitative CAD parameters between the two MRI systems (Table A2).

### 3.2. Invasive Disease-Free Survival

**Figure 2:**
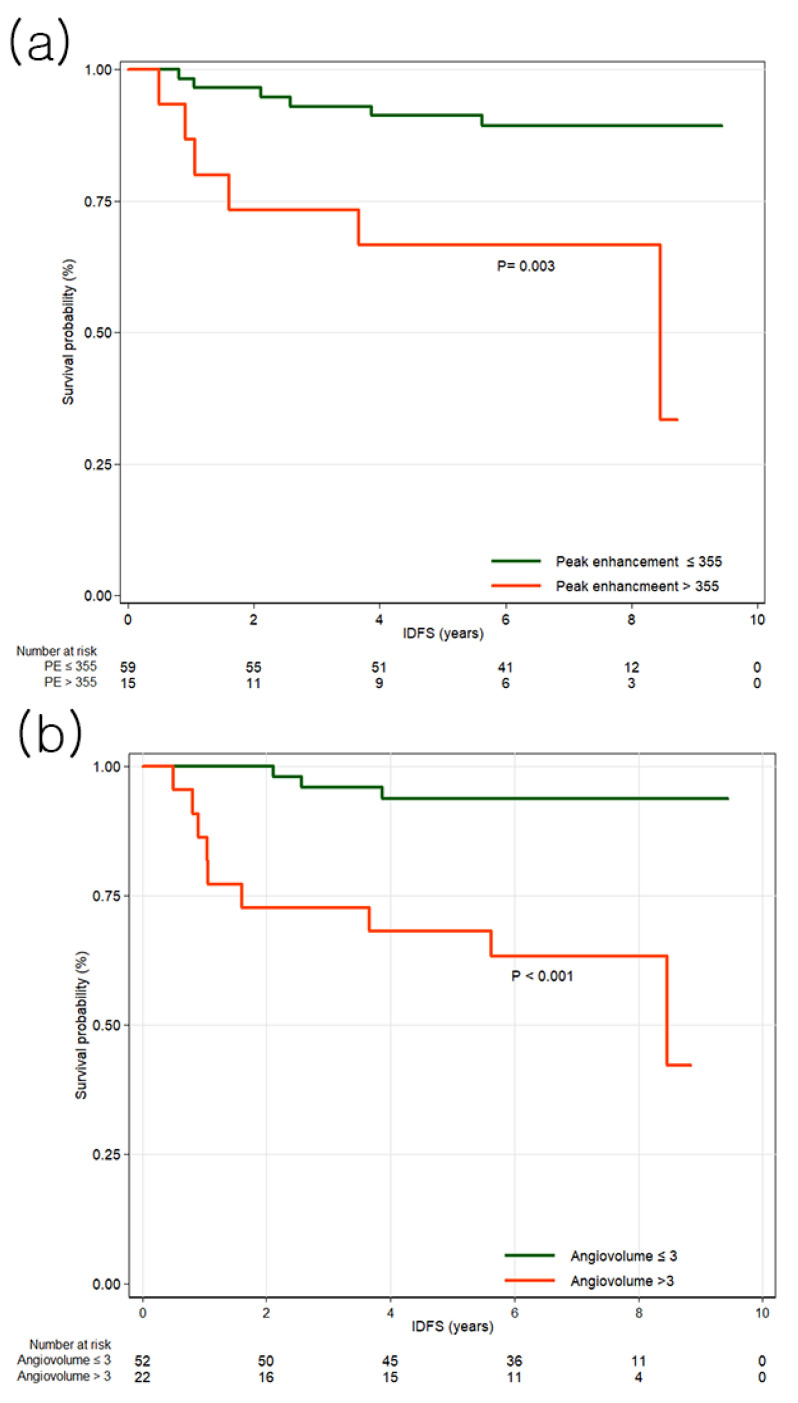
Kaplan–Meier survival curves for invasive disease-free survival (IDFS) by high and low peak enhancement and angiovolume groups. (**a**) The survival curves demonstrate that peak enhancement greater than 355% is associated with IDFS. (**b**) The survival curves demonstrate that angiovolume greater than 3 mL is associated with IDFS.

**Figure 3:**
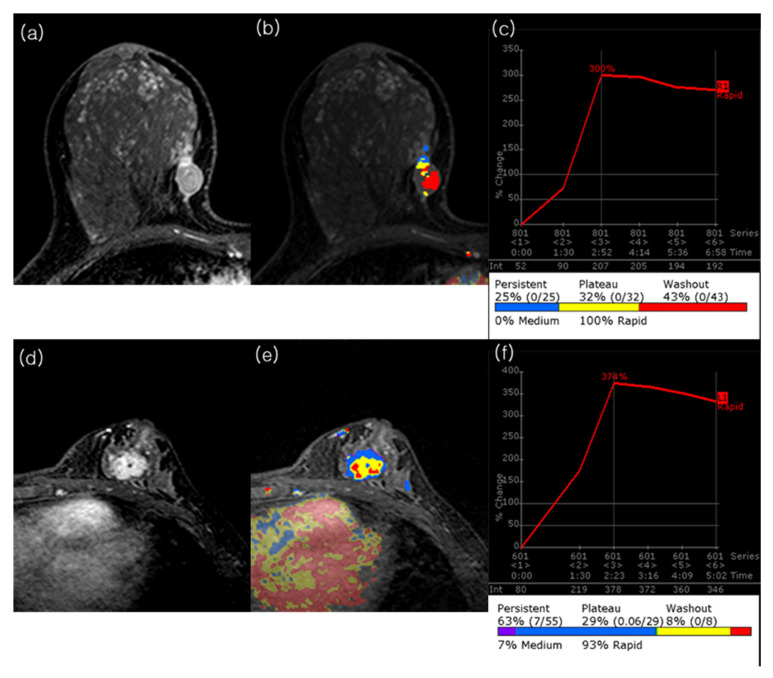
Representative preoperative MRI cases of TNBC with contrasting angiovolume and outcomes. (**a**–**c**) A 40-year-old woman without recurrence during follow-up. Peak dynamic image shows an irregular, rim enhancing 1.6 × 1 × 1.6 cm (calculated tumor volume = 1.34 cm^3^) mass in the right breast. The CAD color map demonstrates prominent intratumoral areas below the threshold, corresponding to an angiovolume of 1.1 cm^3^. The automatically generated portfolio indicates a peak enhancement of 300%. The pathologic tumor size was 2.0 cm, with no axillary lymph node metastasis. (**d**–**f**) A 39-year-old woman with ipsilateral breast cancer and axillary nodal recurrence at 12.7 months after surgery, followed by cervical and mediastinal lymph node metastases at 52.7 months. Peak dynamic image shows an irregular, heterogeneously enhancing 2.4 × 1.8 × 2.4 cm (calculated tumor volume = 5.43 cm^3^) mass in the left breast. The CAD color map demonstrates nearly complete tumor enhancement, corresponding to an angiovolume of 5.4 cm^3^. The portfolio indicates a peak enhancement of 374%. The initial pathologic tumor size was 2.0 cm, with axillary lymph node metastasis.

No clinicopathologic features were significantly associated with IDFS (Table A1). In univariable analysis of MRI features, peak enhancement (HR, 1.30; 95% CI, 1.02–1.66; *p* = 0.033), angiovolume (HR, 1.72; 95% CI, 1.23–2.41; *p* = 0.002), maximum 3D diameter (HR, 1.31; 95% CI, 1.06–1.62; *p* = 0.013), and calculated tumor volume (HR, 1.08; 95% CI, 1.01–1.16; *p* = 0.017) were significantly associated with IDFS (Table A3). In multivariable analysis, peak enhancement (HR, 1.40; 95% CI, 1.06–1.84; *p* = 0.019) and angiovolume (HR, 2.86; 95% CI, 1.26–6.47; *p* = 0.012) remained independently associated with IDFS (Table 1). Cutoff values were determined using the maximally selected log-rank statistic. For IDFS, the optimal cutoff values were 355% for peak enhancement (*p* = 0.003) and 3 mL for angiovolume (*p* < 0.001). Patients were stratified accordingly, and Kaplan–Meier survival curves are presented in Figure 2. Notably, tumors with larger non-enhancing portions within the angiovolume demonstrated better survival compared with those with minimal non-enhancing areas (Figure 3).

### 3.3. Distant Metastasis Free Survival

**Figure 4:**
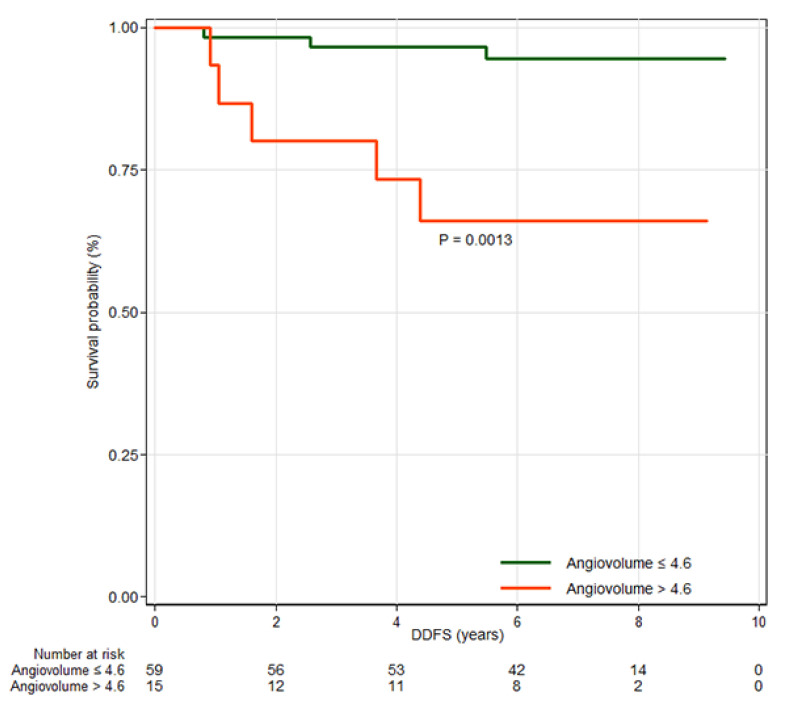
Kaplan–Meier survival curves for distant metastasis-free survival (DDFS) by high and low angiovolume groups. The curves demonstrate that angiovolume greater than 4.6 mL is associated with DDFS.

Among clinicopathologic features, axillary lymph node dissection (HR, 6.19; 95% CI, 1.46–26.19; *p* = 0.013), larger tumor size (HR, 1.97; 95% CI, 1.03–3.76; *p* = 0.040), positive axillary lymph node metastasis (HR, 5.83; 95% CI, 1.45–23.40; *p* = 0.013), and lymphovascular invasion (HR, 7.87; 95% CI, 1.59–39.04; *p* = 0.012) were significantly associated with worse DDFS (Table A4). Among MRI features, peak enhancement (HR, 1.42; 95% CI, 1.08–1.85; *p* = 0.012) and angiovolume (HR, 1.76; 95% CI, 1.16–2.68; *p* = 0.008) were significantly associated with DDFS (Table A5). In multivariable analysis, angiovolume per 5 mL increase (HR, 2.47; 95% CI, 1.28–4.78; *p* = 0.007) remained independently associated with DDFS (Table 2). The optimal cutoff value for angiovolume was 4.6 mL (*p* = 0.0013), determined using the maximally selected log-rank statistic. Patients were stratified into two groups based on this cutoff, and Kaplan–Meier survival curves are shown in Figure 4.

## 4. Discussion

This study identified that among various clinicopathologic and MRI features, peak enhancement and angiovolume were independently associated with worse IDFS outcomes in patients with primary operable TNBC. Furthermore, angiovolume was also independently associated with worse DDFS outcomes.

Previous studies have shown that peak enhancement is associated with disease-specific survival (DSS) and DDFS in TNBC [21,22]. Investigations including all breast cancer subtypes also demonstrated associations between high peak enhancement and poor survival outcomes [15,18,19,27,28]. Although the definition of peak enhancement varies, these studies share the concept of quantifying contrast concentration in both intravascular and extravascular interstitial spaces during the early phase of DCE-MRI. The DCE-MRI contrast enhancement principle, particularly in breast cancer imaging, is based on the increased vascularity and permeability of tumor vasculature due to angiogenesis [29,30]. Tumor angiogenesis is a central hallmark of aggressive breast cancers; intratumoral hypoxia often induces hypoxia-inducible factor -1α activation and subsequent upregulation of vascular endothelial growth factor, promoting the formation of aberrant microvasculature [31]. This angiogenic activity directly leads to increased microvessel density (MVD). Consequently, higher peak enhancement reflects this increased MVD and vascular permeability, indicating a more aggressive tumor with better blood supply, potentially reflecting high tumor grade, metastatic potential and poor prognosis [32,33,34]. Recent studies have shown that high peak enhancement is correlated with low tumor-infiltrating lymphocytes (TIL) levels in HER2-positive tumors, and although the association is not statistically significant, a similar trend has been observed in TNBC [35,36]. The coexistence of high peak enhancement and low TIL levels may reflect the tumor’s abnormal vasculature, which hinders effective immune cell infiltration [37]. The observed association between peak enhancement and IDFS in our cohort highlights its potential as a comprehensive imaging-based prognostic marker related to, but not limited to, MVD.

Tumor size and nodal status are major prognostic factors in breast cancer [38]. However, in TNBC, pathologic tumor size has been reported to be a relatively poor predictor of axillary lymph node metastasis and survival [3,39,40], suggesting a hematogenous spread pattern that allows for rapid systemic dissemination independent of local lymphatic spread. A recent study found that 3D pathologic tumor volume is an independent prognostic factor for IDFS even after controlling for tumor size and nodal involvement [41]. Tumor volume provides a simple understanding of tumor burden. Angiovolume represents the volumetric size of the highly enhancing portion of the tumor. It excludes intratumoral areas with weak or no enhancement, which may correspond to hypovascular areas, hemorrhage, cysts, fibrosis, or necrosis. Angiovolume was identified as a predictor of axillary lymph node metastasis in TNBC patients [42]. In multivariable analysis of primary operable TNBC, angiovolume was independently associated with DDFS, whereas pathologic tumor size was significant only in univariate analysis. Even when the tumor sizes were similar, tumors with larger hypervascular portions showed poorer survival than those with smaller hypervascular areas. In contrast, other MRI features, such as maximum 3D diameter (longest lesion diameter) and calculated tumor volume (which does not exclude weak or non-enhancing tissue), were not associated with IDFS or DDFS. The hypervascular portion in TNBC may be a more accurate indicator of patient prognosis.

Breast cancer subtypes exhibit distinct imaging characteristics, underscoring the need for subtype-specific approaches when analyzing MRI features [24,31]. In this study, high peak enhancement and high angiovolume were found to be correlated with patient poor prognosis. Peak enhancement has been reported as a poor prognostic factor in all breast cancer subtypes [15,16,17,18,19,35,36]. However, angiovolume was only correlated with prognosis in TNBC patients [21,42]. These findings suggest that imaging biomarkers may need to be subtype-specific for breast cancer. While kinetic heterogeneity has been reported as a predictor of DDFS in cohorts including all breast cancer subtypes, it was not significant in our TNBC population, again supporting the need for subtype-specific analyses [18].

Multiparametric studies, including T2WI, T1WI and DWI parameters, have shown variable results in predicting breast cancer patient outcomes [43,44,45]. The higher volume fraction of extracellular space per unit volume of tissue and higher volume fraction with rapid enhancement followed by a persistent curve type have been correlated with worse survival outcome [21,22]. These findings are thought to indicate higher stromal content, which has been linked to poor prognosis, particularly in TNBC. The evaluation of these features requires labor-intensive region of interest drawing and specialized programs, which are difficult to integrate into busy clinical settings. However, there are also metrics, such as the volume fraction with more than 100% initial enhancement rate followed by a persistent curve type, that can be extracted using CAD. This enables the analysis of stromal-associated kinetic characteristics as well. While standardizing imaging biomarkers is challenging, utilizing CAD systems, which can maintain consistency in measurements, makes it more feasible. Exploring and utilizing multiparametric features and stromal-related kinetic characteristics, which can be quickly and easily calculated through commercially available software, is essential for promoting the broader clinical application of imaging biomarkers in precision oncology.

Angiovolume and peak enhancement could potentially be integrated into post-treatment follow-up workflows to assist in risk assessment and guide the intensity of surveillance for TNBC patients. By evaluating these biomarkers during preoperative MRI, clinicians can identify patients who may require more intensive or less frequent surveillance. Radiologists should interpret screening mammography or other surveillance imaging with caution for patients with high angiovolume or peak enhancement, which may help ensure timely intervention and minimize treatment delays.

Our study had several limitations. First, it was a single-institutional study using a single-vendor MRI and CAD system. Although CAD systems offer versatility, their results depend heavily on input imaging data. Variability in acquisition protocols and scanner strength may affect reproducibility. As a single-institution study, there is also a potential for selection bias. Additionally, while the CAD system provides consistent results, size adjustments were possible in a stepwise manner. This process could introduce observer-related variability, as the decision to include or exclude certain areas may vary depending on the reviewer’s judgment. Nevertheless, the stepwise size adjustment is limited, and since most cases do not require adjustments, the variation is expected to be minimal. Second, this was a retrospective study with a small number of patients and survival events, an in particular only eight DDFS events, which requires cautious interpretation [46]. The limited number of events may limit statistical power and increase the risk of overfitting in multivariable models. Therefore, our findings should be considered preliminary tools for risk stratification. To validate the prognostic value of CAD-derived MRI features and ensure their generalizability, external and prospective validation is needed. Incorporating larger and more diverse patient cohorts with variable-stage lesions from multiple centers would allow for more robust statistical analyses and further support the clinical applicability and scalability of these biomarkers.

In conclusion, preoperative CAD-derived MRI features, particularly peak enhancement and angiovolume, were significantly associated with IDFS in TNBC patients, while angiovolume alone was associated with DDFS. These findings reinforce the potential of CAD-derived MRI biomarkers as useful tools for risk stratification in TNBC. However, further validation through larger, prospective studies is essential to confirm their clinical applicability.

## Figures and Tables

**Table 1:** Multivariable analysis of features associated with invasive disease-free survival.

Variable	Hazard Ratio	95% Confidence Interval	*p* Value
Peak enhancement, per increase of 100%	1.40	1.06–1.84	0.019
Angiovolume, per increase of 5 mL	2.86	1.26–6.47	0.012
Maximum 3D diameter (cm)	0.68	0.27–1.69	0.404
Calculated tumor volume, per increase of 5 cm^3^	1.01	0.81–1.27	0.898

**Table 2:** Multivariable analysis of features associated with distant metastasis-free survival.

Variable	Hazard Ratio	95% Confidence Interval	*p* Value
Tumor size	0.66	0.27–1.62	0.368
Type of lymph node dissection			
Sentinel dissection	1		
Axillary dissection	1.27	0.13–12.48	0.840
Axillary node metastasis			
Negative	1		
Positive	2.83	0.32–24.85	0.347
Lymphovascular invasion			
Negative	1		
Positive	10.08	0.86–118.03	0.066
Peak enhancement, per increase of 100%	1.31	0.93–1.85	0.123
Angiovolume, per increase of 5 mL	2.47	1.28–4.78	0.007

## Data Availability

The data presented in this study are available on reasonable request from the corresponding author. The data are not publicly available due to privacy and ethical restrictions.

## Abbreviations

The following abbreviations are used in this manuscript:

TNBC	triple-negative breast cancer
ER	estrogen receptors
PR	progesterone receptors
HER2	human epidermal growth factor receptor type 2
DCE	Dynamic contrast-enhanced
CAD	computer-assisted diagnosis
IDFS	invasive disease-free survival
DDFS	distant metastasis-free survival
IHC	immunohistochemistry
T2WI	T2-weighted imaging
T1WI	T1-weighted imaging
SPAIR	spectral attenuated inversion recovery
TR	repetition time
TE	Echo time
FOV	Field of view
ETL	Echo train length
SI	Signal intensity
HR	hazard ratio
CI	confidence interval
BCS	Breast-Conserving Surgery
DSS	Disease-Specific Survival
MVD	Microvessel Density
TIL	Tumor-Infiltrating Lymphocytes

## Appendix A

**Table A1:** Univariable analysis of clinicopathologic features associated with invasive disease-free survival.

	All Patients (*n* = 74)	Patient Without Invasive Cancer (*n* = 62)	Patient with Invasive Cancer (*n* = 12)	Hazard Ratio	95% Confidence Interval	*p* Value
Age (years) †	51 ± 11.7	51.4 ± 12.1	48.9 ± 9.5	0.99	0.94–1.04	0.66
Type of surgery						
BCS	62 (83.8)	53 (85.5)	9 (75)	0.49	0.13–1.81	0.286
Mastectomy	12 (16.2)	9 (14.5)	3 (25)	1		
Type of Axillary Dissection						
Sentinel dissection	66 (89.2)	57 (91.9)	9 (75)	1		
Axillary dissection	8 (10.8)	5 (8.1)	3 (25)	3.55	0.94–13.44	0.062
Adjuvant chemotherapy						
No	3 (4.1)	3 (4.8)	0 (0)	1		
Yes	71 (96)	59 (95.2)	12 (100)	0.61	0.08–77.96	0.748 *
Adjuvant radiation therapy						
No	11 (14.9)	9 (14.5)	2 (16.7)	1		
Yes	63 (85.1)	53 (85.5)	10 (83.3)	0.77	0.17–3.52	0.737
Tumor size (cm) †	1.8 ± 0.8	1.7 ± 0.7	2.2 ± 1.3	1.67	0.94–2.95	0.078
Axillary node metastasis						
Negative	62 (83.8)	54 (87.1)	8 (66.7)	1		
Positive	12 (16.2)	8 (12.9)	4 (33.3)	3.3	0.96–11.31	0.057
Histologic grade						
1 or 2	15 (20.3)	13 (21)	2 (16.7)	1		
3	59 (79.7)	49 (79)	10 (83.3)	1.31	0.29–5.99	0.730
Lymphovascular invasion						
Negative	53 (71.6)	47 (75.8)	6 (50)	1		
Positive	21 (28.4)	15 (24.2)	6 (50)	2.75	0.88–8.54	0.081
Ki-67 index						
Low (<20%)	11 (14.9)	9 (14.5)	2 (16.7)	1		
High (>20%)	63 (85.1)	53 (85.5)	10 (83.3)	1.03	0.22–4.75	0.968
P53 status						
Negative	28 (27.8)	23 (37.1)	5 (41.7)	1		
Positive	46 (62.2)	39 (62.9)	7 (58.3)	0.82	0.26–2.59	0.730
EGFR status						
Negative	53 (71.6)	44 (71)	9 (75)	1		
Positive	21 (28.4)	18 (29)	3 (25)	0.8	0.22–2.95	0.736
CK5/6 status						
Negative	21 (28.4)	18 (29)	3 (25)	1		
Positive	53 (71.6)	44 (71)	9 (75)	1.16	0.32–4.3	0.819
Basal-like tumor						
Negative	18 (24.3)	15 (24.2)	3 (25)	1		
Positive	56 (75.7)	47 (75.8)	9 (75)	0.94	0.25–3.46	0.921

Note. BCS Breast conserving surgery HR Hazard ratio, CI confidence interval. Except where indicated, data are numbers of patients, with percentages in parentheses. * Cox with firth correction. † Data are means ± standard deviations.

**Table A2:** Comparison of quantitative CAD parameters between the two MRI Systems (Achieva vs. Ingenia).

Variable	Achieva (*n* = 63)	Ingenia (*n* = 11)	*p* Value
CAD parameters			
Peak enhancement (%)	290.1 ± 170.3	252.7 ± 162.7	0.502
Angiovolume (mL)	3.7 ± 5.2	2.8 ± 3.6	0.622
Maximum diameter (cm)	2.3 ± 1.5	2.2 ± 1.1	0.766
Calculated Volume (cm^3^)	7.2 ± 18.5	4.2 ± 5.6	0.602
Early enhancement pattern †			
%V of medium	23.8 ± 29.6	20.5 ± 8.2	0.735
%V of fast	76.3 ± 29.6	79.5 ± 27.2	0.732
Delay enhancement pattern †			
%V of persistent	53.4 ± 27.6	49.6 ± 36.9	0.691
%V of plateau	32.7 ± 17.3	34.8 ± 23.5	0.719
%V of washout	14.0 ± 18.1	15.5 ± 19.7	0.807
Kinetic heterogeneity	0.677 ± 0.276	0.570 ± 0.249	0.230

Note. CAD—computer-assisted diagnosis, %V—percentage volume; † Data are means ± standard deviations.

**Table A3:** Univariable analysis of MRI features associated with invasive disease-free survival.

Variable	All Patients (*n* = 74)	Patient Without Invasive Cancer (*n* = 62)	Patient with Invasive Cancer (*n* = 12)	Hazard Ratio	95% Confidence Interval	*p* Value
T2 weighted image						
Peritumoral edema						
Negative	29 (39.2)	24 (28.7)	5 (41.7)	1		
Positive	45 (60.8)	38 (61.3)	7 (58.3)	0.89	0.28–2.81	0.845
Intratumoral T2 high SI						
Negative	35 (47.3)	32 (51.6)	3 (25)	1		
Positive	39 (52.7)	30 (48.4)	9 (75)	2.87	0.77–10.65	0.115
CAD parameters						
Peak enhancement, per increase of 100% †	284.5 ± 168.6	266.6 ± 148.8	377.3 ± 233.8	1.3	1.02–1.66	0.033
Angiovolume, per increase of 5 mL †	3.5 ± 5.0	2.6 ± 3.5	8.2 ± 8.1	1.72	1.23–2.41	0.002
Maximum diameter (cm) †	2.3 ± 1.5	2.1 ± 1.1	3.5 ± 2.4	1.31	1.06–1.62	0.013
Calculated tumor Volume, per increase of 5 cm^3^ †	6.8 ± 17.2	3.7 ± 5	22.9 ± 38.5	1.08	1.01–1.16	0.017
Early enhancement pattern †						
%V of medium	23.3 ± 29.1	23.4 ± 29.3	22.9 ± 29.2	1	0.98–1.02	0.86
%V of fast	76.7 ± 29.1	76.7 ± 29.3	77.2 ± 29.2	1	0.98–1.02	0.86
Delay enhancement patter †						
%V of persistent	52.9 ± 28.9	53.8 ± 28.4	47.8 ± 32.2	0.99	0.96–1.01	0.356
%V of plateau	33 ± 18.1	33.1 ± 18.7	32.2 ± 15.3	1	0.97–1.03	0.965
%V of washout	14.2 ± 18.2	13.1 ± 17	20.1 ± 23.6	1	0.98–1.02	0.812
Kinetic heterogeneity †	0.661 ± 0.273	0.656 ± 0.279	0.687 ± 0.247	1.33	0.15–11.61	0.796

Note. SI—signal intensity, CAD—computer-assisted diagnosis, %V—percentage volume; except where indicated, data are numbers of patients, with percentages in parentheses. † Data are means ± standard deviations.

**Table A4:** Univariable analysis of clinicopathologic features associated with distant metastasis-free survival.

	All Patients (*n* = 74)	Patient Without Distant Metastasis (*n* = 66)	Patient with Distant Metastasis (*n* = 8)	Hazard Ratio	95% Confidence Interval	*p* Value
Age (years) †	51 ± 11.7	50.9 ± 11.9	51.8 ±10.2	1.01	0.95–1.07	0.823
Type of surgery						
Breast conserving surgery	62 (83.8)	56 (84.9)	6 (75)	0.48	0.1–2.39	0.371
Mastectomy	12 (16.2)	10 (15.2)	2 (25)	1		
Type of Axillary Dissection						
Sentinel dissection	66 (89.2)	61 (92.4)	5 (62.5)	1		
Axillary dissection	8 (10.8)	5 (7.6)	3 (37.5)	6.19	1.46–26.19	0.013
Adjuvant chemotherapy						
No	3 (4.1)	3 (4.6)	0 (0)	1		
Yes	71 (96)	63 (95.5)	8 (100)	0.42	0.05–53.97	0.595 *
Adjuvant radiation therapy						
No	11 (14.9)	10 (15.2)	1 (12.5)	1		
Yes	63 (85.1)	56 (84.9)	7 (87.5)	1.06	0.13–8.62	0.956
Tumor size (cm) †	1.8 ± 0.8	1.7 ± 0.7	2.3 ± 1.5	1.97	1.03–3.76	0.040
Axillary node metastasis						
Negative	62 (83.8)	58 (87.9)	4 (50)	1		
Positive	12 (16.2)	8 (12.1)	4 (50)	5.83	1.45–23.4	0.013
Histologic grade						
1 or 2	15 (20.3)	14 (21.2)	1 (12.5)	1		
3	59 (79.7)	52 (78.8)	7 (87.5)	1.96	0.24–15.92	0.530
Lymphovascular invasion						
Negative	53 (71.6)	51 (77.3)	2 (25)	1		
Positive	21 (28.4)	15 (22.7)	6 (75)	7.87	1.59–39.04	0.012
Ki-67 index						
Low (<20%)	11 (14.9)	10 (15.2)	1 (12.5)	1		
High (>20%)	63 (85.1)	56 (84.9)	7 (87.5)	1.36	0.17–11.07	0.773
P53 status						
Negative	28 (27.8)	25 (37.9)	3 (37.5)	1		
Positive	46 (62.2)	41 (62.1)	5 (62.5)	1.02	0.24–4.26	0.982
EGFR status						
Negative	53 (71.6)	47 (71.2)	6 (75)	1		
Positive	21 (28.4)	19 (28.8)	2 (25)	0.83	0.17–4.1	0.816
CK5/6 status						
Negative	21 (28.4)	19 (28.8)	2 (25)	1		
Positive	53 (71.6)	47 (71.2)	6 (75)	1.28	0.26–6.32	0.766
Basal-like tumor type						
Negative	18 (24.3)	16 (24.2)	2 (25)	1		
Positive	56 (75.7)	50 (75.8)	6 (75)	1.02	0.21–5.04	0.983

Note. CAD—computer-assisted diagnosis, %V—percentage volume; except where indicated, data are numbers of patients, with percentages in parentheses. * Cox with firth correction. † Data are means ± standard deviations.

**Table A5:** Univariable analysis of MRI features associated with distant metastasis-free survival.

Variable	All Patients (*n* = 74)	Patient Without Distant Metastasis (*n* = 66)	Patient with Distant Metastasis (*n* = 8)	Hazard Ratio	95% Confidence Interval	*p* Value
T2 weighted image						
Peritumoral edema						
Negative	29 (39.2)	26 ± 39.4	3 ± 37.5	1		
Positive	45 (60.8)	40 ± 60.6	5 ± 62.5	1.04	0.25–4.33	0.962
Intratumoral T2 high SI						
Negative	35 (47.3)	34 (51.5)	1 (12.5)	1		
Positive	39 (52.7)	32 (48.5)	7 (87.5)	6.72	0.83–54.63	0.075
CAD parameters						
Peak enhancement, per increase of 100% †	284.5 ± 168.6	267.0 ± 146.6	429 ± 264.9	1.42	1.08–1.85	0.012
Angiovolume, per increase of 5 mL †	3.5 ± 5.0	3.0 ± 4.3	8.1 ± 7.8	1.76	1.16–2.68	0.008
Maximum diameter (cm) †	2.3 ± 1.5	2.2 ± 1.4	3.2 ± 1.6	1.28	0.96–1.7	0.088
Calculated Volume, per increase of 5 cm^3^ †	6.8 ± 17.2	5.7 ± 16.8	15.2 ± 19.4	1.07	0.97–1.18	0.182
Early enhancement pattern †						
%V of medium	23.3 ± 29.1	23.8 ± 29.3	19.3 ± 29.1	0.99	0.97–1.02	0.610
%V of fast	76.7 ± 29.1	76.3 ± 29.3	80.8 ± 29.1	1.01	0.98–1.03	0.610
Delay enhancement pattern †						
%V of persistent	52.9 ± 28.9	53.6 ± 28.5	47 ± 33.6	0.98	0.94–1.01	0.189
%V of plateau	33 ± 18.1	32.9 ± 18.4	33.5 ± 17	1.01	0.97–1.05	0.587
%V of washout	14.2 ± 18.2	13.6 ± 17.8	19.8 ± 22.3	0.99	0.97–1.02	0.659
Kinetic heterogeneity †	0.661 ± 0.273	0.658 ± 0.272	0.685 ± 0.299	1.05	0.80–1.36	0.743

Note. CAD—computer-assisted diagnosis, %V—percentage volume; except where indicated, data are numbers of patients, with percentages in parentheses. † Data are means ± standard deviations.
